# Editorial: Engineering the Plant Biofactory for the Production of Biologics and Small-Molecule Medicines—Volume 2

**DOI:** 10.3389/fpls.2022.942746

**Published:** 2022-07-07

**Authors:** Domenico De Martinis, Inga Isabel Hitzeroth, Ryo Matsuda, Natacha Soto Pérez, Eugenio Benvenuto

**Affiliations:** ^1^Italian National Agency for New Technologies, Energy and Sustainable Economic Development (ENEA), Rome, Italy; ^2^Biopharming Research Unit, University of Cape Town, Cape Town, South Africa; ^3^Graduate School of Agricultural and Life Sciences, The University of Tokyo, Tokyo, Japan; ^4^Plant Biotechnology Department, Center for Genetic Engineering and Biotechnology (CIGB), Havana, Cuba

**Keywords:** biopharmaceuticals, recombinant protein, genetic engineering, transient expression, plant biofactory, antibodies, vaccines

The transfer of genes into plants, that was achieved in the early 80's, paved the way for the exploitation of the potential of plant genetic engineering, to add novel agronomic traits and/or to design plants as factories for high added value molecules.

“Molecular Farming” was a term coined in reference to such agricultural applications, and major crops like maize and tobacco were originally used for pharma applications. It was since these early studies on plant gene transfer that the scientific community interpreted the technology not only for improving plant performance, as an extension of the plant breeding concept but rather to produce new products, to use the plant as a biofactory for novel designed molecules.

The concept of the “green biofactory” implies different advantages over the typical cell factories based on animal cell or microbial cultures alone when considering the investment and managing costs of fermenters. Although yield, stability, and quality of the molecules may vary among different heterologous systems and plants are competitive on a case-to-case basis, still the “plant biofactory” attracts scientists and technologists for the challenging features of low production cost, product safety, and easy scale-up. The rush to develop a vaccine and the need for fast scale-up production in the years of the COVID-19 pandemic highlighted how a plant biofactory may be useful for global-scale production of large amounts of medicals.

Therefore, in this Research Topic we have tried to gather (again) together the scientific community working on the concept of plant biofactories, as successfully achieved in 2016 (De Martinis et al.). The topic focused on exploring the type of molecules that are currently studied and produced in plants and the approaches to obtain pharmaceutical proteins, medical diagnostics proteins, and vaccine antigens, at an industrial scale. We devoted the work to recent scientific progress in the areas of plant-produced antibodies and vaccines, medicals and diagnostics; protein design for heterologous production in plant biofactories; synthetic biology applied to agriculture; biorefinery, biochemical, and molecular level studies.

The display of studies of this “volume II” gathers together 103 authors from the USA, Canada, Europe, South Africa, South Korea, Thailand, India, and Japan, that approached the complexity of producing desired molecules in plants and plant cells, covering the topic from engineering, to methods to increase the quality and quantity of the desired molecule.

Several papers in this topic described methods to produce diagnostic or vaccines in plants, including COVID-19 related products; efforts to demonstrate the use of plants to produce effective yet affordable vaccines and fast production of viral antigens, which are required by the industry in high amounts also for serological assays, were made (Schwestka et al.; Siriwattananon et al.); approaches on how to better produce molecules of interest is further explored, with the production of functional antibodies already in the market, such as the Denosumab, used in therapy for osteoporosis (Boonyayothin et al.), and the Pembrolizumab for cancer immunotherapy (Phakham et al.), and the production of chimaeric antibody functional in binding and neutralizing enterohemorrhagic *Escherichia coli* (Chin-Fatt and Menassa).

Plant biofactory, as for others biorefineries, also requires strategies to increase quantity and quality of the molecule of interest; this is the case of a (glyco-) engineered plant line to be used for the production of a functional enzyme β-glucocerebrosidase, for Gaucher disease treatment (Uthailak et al.), and potentially for other pharmaceutical proteins, especially mannose receptor targeted protein (Sariyatun et al.), and the production of heterologous viral glycoproteins in plants with authentic human-like glycosylation (Margolin et al.). Also the use of the B1 domain of Streptococcal protein G (GB1) proved how a multi-functional domain used in recombinant proteins in plants, could both stabilize a chimeric protein and facilitate its detection (Song et al.).

In addition to the quality of a plant produced heterologous molecule, speed of production and yield are important; transgenic plants transformed with a positive cell cycle regulator gene *At-CycD2* resulted in enhanced recombinant protein yield (Kopertekh and Reichardt), cell-free biofactories can be of use for the production of proteins and metabolites within a few hours or days (Buntru et al.), while modifying anthocyanins biosynthesis in plants may represent a strategy to obtain resistance to ionizing radiation and anti-oxidant properties during cultivation in space (Massa et al.). This latest work recalls and binds to the issue of improving the plant biofactory performance to another important aspect, described in the 2020 Front. Plant Science Res. Topic *Next Generation Agriculture: Understanding Plant Life for Food, Health, and Energy*, that is, the necessity to cultivate in non-traditional environments such as indoor urban scenarios, extremely cold or dry areas or even in space, either on orbit around the planet or during space traveling.

The production of any sort of molecules in plants has a great potential, in terms of quality, quantity and economy. This is not restricted to recent approaches to tackle the COVID-19 pandemics, but dates back for decades before and has been suggested to be valuable for rapid production and scale-up already, e.g., in the case of SARS “1” (De Murtas et al.). Once engineered, a plant is among the cheapest and easiest eukaryotic systems to be bred with simple know-how, using nutrients, water and light, and global knowledge of agriculture is well-established for centuries.

“Farming for Pharming” biologics and small-molecule medicines is a challenging area of plant biotechnology that may break the limits of current standard production technologies. The success of fighting Ebola with plant-made antibodies put the spotlight on the enormous potential of next-generation herbal medicines made especially in the name of the guiding principle of reduction of costs, hence reduction of disparities of health rights and as a tool to guarantee adequate health protection in developing countries.

Nevertheless, the recent global sanitary emergency, caused by the COVID-19 pandemic, suggest that the decision makers are not familiar, not at ease, or at least not convinced, by the opportunity of using the plant as easy and scalable biorefinery; the race to the COVID-19 vaccine in most westernized countries ignored the opportunity to work on such platform, neither to promote it as alternative production system to support vaccination campaigns, and/or therapy and diagnostic, in less favorite countries, that would have been helpful during the containment phase when the disease emerged. A similar cold-shoulder has been given to non-EU and non-USA vaccines, as in the case of Russian and Cuban production.

The ability of plants to produce heterologous pharmaceutical proteins has been demonstrated in hundreds of proof-of-principle studies and in a growing number of clinical trials, with a small number of products reaching the market as approved biologics or medical devices (Lobato Gómez et al., 2021), but molecular farming has not overcome the barriers of industry inertia and regulatory restraints. Broad markets and producers as e.g., USA and EU, show disharmony (Case Studies in Agricultural Biosecurity, n.d; European Commission, n.d). The production of biopharmaceutical products through plants lacks specific approved guidelines on the points to consider for the manufacture and application of these products. In this sense, the implementation of new manufacturing processes and quality systems using quality risk management is recognized as something of prime importance in the current pharmaceutical industry. In a thorough review published in Frontiers in 2020 it was discussed how molecular farming could provide practical solutions to address the COVID-19 outbreak in Italy (Lico et al., 2020), that was the first country in Europe to face a large-scale COVID-19 outbreak and it is one of the hardest-hit countries in the EU.

Research carried out in Cuba, showed that the application of the FMEA (Frank et al., 2008) approach to design the manufacturing process of a “plantibody” is necessary for the production of vaccines against hepatitis B, and to guarantee the high quality of the vaccine (Mila et al., 2010). Still in Cuba, several research studies demonstrated the capacity for producing vaccines, antibodies (Hernández-Velázquez et al., 2015), and to purify products to the requested quality and yield for industrial production (Ferro et al., 2015). More generally, the production of transgenic crops in Latin America is increasing. Some countries such as Brazil, Argentina, Mexico, Colombia, Cuba, Honduras, and Uruguay have the necessary practice on the subject of biosafety of transgenic crops. Regarding biosafety regulation and progress in Latin America, the regulatory powers of the countries in the region are heterogeneous. Again, no consensus exists on the importance and development of molecular farming across the region. In many cases, the necessary background and skills for evaluating aspects of biosafety and the operation of regulatory systems are missing (Barragán-Ocaña et al., 2019).

In Japan, InterBerry α^®^, a lyophilized powder of transgenic strawberry fruit expressing canine interferon-α to treat canine gingivitis, was approved in 2013. A highly contained factory is used to produce this veterinary pharmaceutical. It comprises the upstream controlled-environment plant production facility with artificial lighting, which follows the domestic law involving the Cartagena Protocol on Biosafety to prevent transgene flow to the outside, and the GMP-compliant downstream processing facility. This type of factory should be a model to manufacture plant-made pharmaceuticals commercially in Japan. On the other hand, biopharmaceuticals for human use have not yet been approved. The requirements are expected to be discussed in detail in the future.

In South Africa, the Biopharming Research Unit (BRU) at the University of Cape Town (UCT), a group at the SA Council for Scientific and Industrial Research (CSIR) and Cape Bio Pharms are the primary three molecular farming research and development teams in the country. There are presently no Good Manufacturing Practice (GMP) facilities in South Africa for plant-produced biopharmaceuticals (Murad et al., 2020) and Cape Bio Pharms, a spin-off company of UCT aimed at commercializing the biotech developed by the BRU, is the only pilot-scale manufacturing facility. In response to the pandemic Cape Bio Pharms expressed antibodies and various regions of the COVID-19 spike protein in plants and some are used in lateral flow devices which have been approved in South Africa by the South African Health Products Regulatory Authority (SAHPRA) for use in South Africa. They plan to build a GMP facility in the near future, which will be a boost for the development of plant-based pharmaceuticals in Africa.

Moreover, one company that has been active in exploiting the plant based platform is *Medicago*, a Canadian biopharmaceutical company, that combined their plant produced COVID-19 vaccine candidate with GSK's pandemic adjuvant and submitted the positive Phase 3 data for regulatory review by Health Canada in December 2021. This vaccine is stable at 4°C and, if authorized, would be the world's first ever plant-based vaccine approved for human use.

Overall, those examples, and the amount of studies developed so far, suggest the great potential for the use of plants, which could be of use for large-scale deployment for plant-produced vaccine and biologics manufacturing. That valuable opportunity happens to be hampered by the heterogeneity of rules and lack of common understanding, and an inability to achieve a proper technological transfer and reach out to the market with continuity, although the first steps of the molecular farming technology were made 40 years ago already.

A possible explanation of such under exploitation of this type of production platform may be the lack of reliability (legislation-wise), as explained, the competitiveness of other well-established systems (bacto-, myco-, animal-based) that hold fast to their market share, and a significant presence of anti- GMO citizen groups, that may ignore how current pharmaceuticals are actually produced. This suggests a failure of the plant science community in communicating such opportunity to the decision makers as well as to the civil society. That leads ultimately to a reflection on how plant science communication should develop, to be able to provide the appropriate information to the society; that would reflect in policy makers to take informed decisions, and citizens to make informed choices.

## Author Contributions

All authors listed have made a substantial, direct, and intellectual contribution to the work and approved it for publication.

## Conflict of Interest

IH declares that she holds shares in Cape Bio Pharms. The remaining authors declare that the research was conducted in the absence of any commercial or financial relationships that could be construed as a potential conflict of interest.

## Publisher's Note

All claims expressed in this article are solely those of the authors and do not necessarily represent those of their affiliated organizations, or those of the publisher, the editors and the reviewers. Any product that may be evaluated in this article, or claim that may be made by its manufacturer, is not guaranteed or endorsed by the publisher.
